# Zero-derivation in Korean: the effect of covert structure in real-time processing

**DOI:** 10.3389/fpsyg.2023.1230927

**Published:** 2023-12-13

**Authors:** Nayoun Kim, Ziying Li, Seonghyeon Byeon, Chaejin Lee

**Affiliations:** Department of English Language and Literature, Sungkyunkwan University, Seoul, Republic of Korea

**Keywords:** categorially ambiguous words, adjectives and verbs, zero derivation, real-time processing, experimental syntax

## Abstract

Korean words like *balgda* ‘bright/become bright’ and *gilda* ‘long/become long’ are categorially ambiguous; they can appear as both adjectives and verbs. Some suggest that these words are listed under separate lexical entries, while others propose that they share one single lexical entry, and that the verb form is morphologically derived from the base adjective through a process called zero derivation. This study presents the results of a real-time experiment that investigates whether these words involve zero derivation and if so, how zero derivation may affect the real-time processing of these words. Our findings suggest that the reader recognizes the base adjective and obtains the derived-verb form by virtue of adding a covert category-changing morpheme in real-time sentence processing. This study provides promising evidence of the zero derivation of Korean categorially ambiguous adjectives and verbs, as well as crosslinguistic evidence of the role of covert structure in lexical access.

## 1 Introduction

In Korean, words like *balgda* ‘bright/become bright’ are ambiguous in terms of their categories; they can appear as both adjectives and verbs, as in (1a) and (1b), respectively.^1^

**Table:** 

(1)	a.	Bang-i	*balg*-Ø-da.	
		room-NOM	bright-PRS-DECL	
		‘The room is bright.’		
	b.	Nal-i	*balg*-neun-da.	
		day-NOM	dawn-PRS-DECL	
		‘The day dawns.’		(Song, 2014, p. 348)

This study concerns words that can appear as both adjectives and verbs, and we refer to these words as categorically ambiguous adjectives and verbs. The grammatical categories of these words have been investigated (e.g., Song, 1988; Koo, 2010, 2021; Yang, 2015), with some researchers suggesting that they belong to two different categories listed under separate lexical entries with distinct “forms,” “functions,” and “meanings” (Koo, 2010; Yang, 2015; for English, see Jackendoff, 1976; Lieber, 1980).

An alternative account proposes that these words undergo a process of zero derivation, with the form in one category ([_*ADJ*_ *balgda* ‘bright’]) being the base, and the other ([_*V*_ *balgda* ‘become bright’]) being morphologically derived from the base-category stem by attaching a phonologically unrealized suffix (i.e., zero morpheme) (Song, 1988; Koo, 2021; for English, see Marchand, 1964; Clark and Clark, 1979; Don, 2005; Lukic, 2016; Krasuska and Yoshida, 2019; Lukic et al., 2023; also see the discussion from the perspective of distributed morphology, which explores the syntactic structure of a lexical item; Halle and Marantz, 1993, 1994; Harley and Noyer, 1999; Harley, 2011, 2014).

Whether categorially ambiguous words involve the zero-derivation process with a covert morpheme has been considerably discussed over the past few decades and is still under debate (cf. Dahl and Fábregas, 2018). This study leverages the previous research suggesting that readers recognize the zero morphemes as morphological units (Lukic, 2016; Krasuska and Yoshida, 2019; Lukic et al., 2023). We investigate whether the covert structure-building process involved in yielding zero-derived categorially ambiguous nouns and verbs observed in English can also be detected in the case of categorially ambiguous verbs in Korean.

Under the hypothesis of zero morphology, the word-internal structure for the base-form word and its zero-derived counterpart should be different, with the latter being morphologically more complex, as shown in the comparison in (2a) and (2b) (Myers, 1984; Krasuska and Yoshida, 2019).

**Table:** 

(2)	a.	word-internal structure for the base form (e.g., [_*ADJ*_ *balgda* ‘bright’])
		ADJ (*balgda* ‘bright’)
		|
		*balgda* ‘bright’
	b.	word-internal structure for the derived form (e.g., [_*V*_ [_*ADJ*_ *balgda* ‘bright’]*-Ø* ‘become’])
		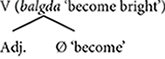
		|
		*balgda* ‘bright’

In this study, we investigate whether only the base form ([_*ADJ*_ *balgda* ‘bright’]; 2a) is represented in the lexicon and the derived form ([_*V*_ *balgda* ‘become bright’]; 2b) is achieved through the addition of covert structure that is built during online sentence comprehension (for English, see Krasuska and Yoshida, 2019; Lukic et al., 2023).

Sentence comprehension involves integrating words into a structure and keeping track of the incomplete input (Gibson, 1998, 2000). For example, in (1), when a nominative noun phrase (NP) such as “*bang*” ‘room’ or “*nal*” ‘day’ appears as an input and is constructed, an incomplete syntactic dependency is formed. The NP needs at least a predicate to complete the sentence structure and interpretation. If more material appears, this will add to the structural complexity because more inputs need to be built, and the incomplete sentence structure needs to be maintained in memory (Gibson, 1998).

Similarly, if categorially ambiguous adjectives and verbs in Korean undergo derivation, the base adjective (input) will be constructed first, and then the zero morpheme must be built to complete the word structure and obtain the adjective-derived verb. In example (1), suppose the reader builds the word structure and incorporates the obtained word form ([_*ADJ*_ *balgda* ‘bright’] in 2a; [_*V*_ *balgda* ‘become bright’] in 2b) into the sentence structure, when encountering a categorially ambiguous word like *balgda* ‘bright/become bright.’ In that case, the additional step in word-internal structure building in the adjective-derived verb (as in (2b) compared to (2a)) would yield concomitant processing costs during real-time sentence processing.

By comparing the processing differences between Korean categorially ambiguous adjectives and verbs, this study aims to explore whether these words may result from zero derivation. We focus on categorially ambiguous adjectives and verbs because adjectives and verbs have overlapping syntactic distribution and are usually distinguished by their morphosyntactic properties in Korean (i.e., derivational suffixes; Sohn, 1999, 2004, p. 229). Additionally, we focus on only one derivational direction (i.e., verbs derived from adjectives), which allows a direct comparison with categorially unambiguous adjectives and verbs.

## 2 Background

### 2.1 Previous experimental studies of the processing of English categorially ambiguous nouns and verbs

Studies on categorially ambiguous nouns and verbs in English have been carried out to support zero derivation and to determine the base category and the derivative from various perspectives (Marchand, 1964; Clark and Clark, 1979; Myers, 1984; Hale and Keyser, 1993, 1997; Borer, 2014). For example, from a morphological perspective, Myers (1984) pointed out that a zero-derived word can undergo a further process of inflection (e.g., [_*N.*_*_*pl*_* [_*N*_ [_*V*_ *walk*]-Ø]-*s*]) but not derivation with a root-selecting suffix due to the intervening zero morpheme (e.g., [_*ADJ*_ [_*N*_ *danger*]-*ous*] vs. *[_*ADJ*_ [_*N*_ [_*V*_ *walk*]-Ø]-*ous*], morpheme ordering principle; Williams, 1981).

Syntactically, it is noted that verb-derived nouns cannot take verbal argument structure (e.g., [_*V*_ *walked*] *the dog for three hours* vs.*the [_*N*_ *walk*] *of the dog for three hours*; Borer, 2014, p. 131), and denominal (noun-derived) location verbs can only be transitive (e.g., *She corralled her horses* vs. *Her horses corralled; Hale and Keyser, 1993, 1997, p. 206). From a semantic perspective, Marchand (1964) pointed out that the analysis of a zero-derived word must be semantically dependent on its base counterpart (e.g., to [_*V*_ *saw*] = to cut something with a [_*N*_ *saw*]).

From the processing perspective, given that structure building incurs processing costs and building a more complex structure involves greater processing costs (Gibson, 1998, 2000), the zero-derived form should be more difficult to process than the base form of a categorially ambiguous word due to its greater morphological complexity, as shown in Lukic et al. (2023).

Lukic et al. (2023) conducted two experiments to investigate how morphological complexity affected processing of categorially ambiguous words in lexical access (Experiment 1) and online sentence processing (Experiment 2). In their Experiment 1 (forced-choice phrasal-completion task), they compared the selection rates and reaction times for the categorially (un)ambiguous nouns and verbs as in (3).

**Table:** 

(3)	Example experimental nouns and verbs used in Lukic et al. (2023)	
a.	(*the/to*) *tray*	(categorially unambiguous noun)
b.	(*the/to*) *eat*	(categorially unambiguous verb)
c.	(*the/to*) *paint*	(categorially ambiguous noun)
d.	(*the/to*) *visit*	(categorially ambiguous verb)
		(Lukic et al., 2023, p. 4 (1))

Conditions A and B were categorially unambiguous nouns and verbs, appearing only with *the* (3a) or *to* (3b), respectively. Conditions C and D presented categorially ambiguous words, where condition C presented words with the noun regarded as the base category, and condition D the verb regarded as the base category. Participants were asked to select between *the* and *to* to complete the phrase as in (3). They observed a “base-category bias” for the categorially ambiguous words: readers selected *the* for categorially ambiguous nouns (*paint*; 3c), and *to* for categorially ambiguous verbs (*visit*; 3d), in a way similar to the categorially unambiguous nouns (*tray*; 3a) and verbs (*eat*; 3b).

The same bias for base category was also found in their Experiment 2 (eye-tracking while reading task). They investigated categorially ambiguous noun/verb pairs in unambiguous contexts like “Rachel needed *the/to* paint since the house looked old” (Lukic et al., 2023, p. 9 (2)), and observed a reading time slowdown at the zero-derived word in both directions (e.g., noun-derived verb [_*V*_ *paint*], verb-derived noun [_*N*_ *visit*]). Based on these results, they suggested that the reader recognized the base category and was sensitive to the morphological complexity in the word structure.

The present study tests how covert structure may impact Korean categorially ambiguous adjectives and verbs in real-time sentence comprehension. The English study looked at nouns and verbs which were all tied up in theories about the representations of “objects” and “actions.” We examine Korean adjectives and verbs, which are subcategorized as “stative predicates” (Sohn, 1999; Kim, 2002; Koo, 2021). Additionally, English and Korean differ greatly in terms of typology. Korean tends to be agglutinative, allowing words to contain multiple morphemes, and each morpheme corresponds to a distinctive syntactic category and semantic meaning. English tends to be fusional, in which one morpheme can carry more than one syntactic and semantic property (Bauke and Roeper, 2012; Payne, 2017; Ramoo, 2021).^2^ Therefore, if there is a zero morpheme that carries a specific syntactic category and semantic meaning as in Korean, we expect a zero morpheme in Korean to exhibit higher sensitivity than in English. If we find a similar effect with a different pair of categories in another language to that noted in previous studies of English for nouns and verbs, this can add crosslinguistic evidence to the role of zero derivation on categorially ambiguous words in real-time processing (Lukic, 2016; Krasuska and Yoshida, 2019; Lukic et al., 2023).

### 2.2 Determining the base form of a categorially ambiguous adjective and verb in Korean

Previous analyses have sought to determine the base category of categorially ambiguous adjectives and verbs in Korean according to their morphosyntactic and semantic properties. For instance, Song (1988) noted that the derived form is semantically constrained compared to its base form, as shown in the comparison in (4a) and (4b).

**Table:** 

(4)	a.	Nal-i/Bang-i/saeg-i/pyojeong-i	
		day-NOM/room-NOM/color-NOM/facial.expression-NOM	
			*balg*-Ø-da.
			bright-PRS-DECL
		‘The day/room/color/face is bright.’	
	b.	Nal-i/*saeg-i/*pyojeong-i	
		day-NOM/*color-NOM/*facial.expression-NOM	
			*balg*-neun-da.
			become.bright-PRS-DECL
		‘The day/*color/*face becomes bright.’	
			(Song, 1988, p. 16, modified)

In example (4a), [_*ADJ*_ *balgda* ‘bright’] can take discourse subjects including “day,” “room,” “color,” and “face.” However, in example (4b), [_*V*_ *balgda* ‘become bright’] can only take “day” as its subject. Therefore, [_*ADJ*_ *balgda* ‘bright’] is considered the base form, as it can take subjects belonging to a larger semantic field.

It is also pointed out that the phonologically unrealized zero morpheme can have semantic realization, adding a level of semantic complexity (Pesetsky, 1996; Harley and Noyer, 1999; Folli and Harley, 2007; Koo, 2021; also see Roeper, 2020 for a discussion of the thematic roles of zero morpheme): [_*ADJ*_ *balgda* ‘bright’] describes the status of an entity, whereas the zero morpheme in the adjective-derived verb [_*V*_ *balgda* ‘bright’-Ø ‘become’] adds a level of meaning indicating a process of the change of status. Moreover, the derivational suffix *-a/eo.jida* ‘become’ can be attached to the adjective stem [_*ADJ*_ *balgda* ‘bright’] to form a verb describing change or degree of the status, but not to the zero-derived verb [_*V*_ *balgda* ‘become bright’], as shown in the comparison in (5a) and (5b).

**Table:** 

(5)	a.	Mudae-ga	*balg*-a.ji-n-da.
		stage-NOM	bright-become-PRS-DECL
		‘The stage lights up.’	
	b.	*Nal-i	*balg*-Ø-a.ji-n-da.
		day-NOM	bright-become-become-PRS-DECL
		‘*The day becomes dawn.’	
			(Koo, 2021, p. 8–9)

The incompatibility of *-a/eo.jida* ‘become’ being attached to [_*V*_ *balgda* ‘become bright’] in (5b) suggests that the zero morpheme realizes the meaning of “becoming.” This is compatible with Myers’s (1984) generalization that a suffix cannot be further attached to a zero-derived word due to the intervening zero morpheme (also see Harley and Noyer, 1999). As a base form, [_*ADJ*_ *balgda* ‘bright’] can take the derivational suffix *-a/eo.jida* ‘become,’ as in (5a), whereas the zero-derived verb [_*V*_ *balgda* ‘become bright’] cannot, as in (5b).

We follow these analyses and investigate the categorially ambiguous words with the adjectives being the base form in this study (Song, 1988; Koo, 2021).

## 3 The experiment: the processing of categorially ambiguous adjectives and verbs in Korean

We conducted a self-paced reading experiment to explore whether and how categorially ambiguous adjectives and verbs in Korean are processed differently. We investigated whether the reader builds the word-internal structure and recognizes the morphological complexity in real-time sentence processing.

### 3.1 Participants

Sixty native Korean speakers (age: 20–40) participated in the experiment. They were each paid ₩2000 as a reward.

### 3.2 Materials and procedure

We employed a 2 × 2 within-subjects design, where *Category* (adjectives vs. verbs) was crossed with *Category Ambiguity* of the words (categorially ambiguous vs. unambiguous). The categorially ambiguous words were adopted from Koo (2021), with adjectives regarded as the base form and verbs as the derived form based on morphosyntactic and semantic analyses. The categorially unambiguous words were those simply used only as adjectives or verbs, according to *Pyojun-Gugeo-Daesajeon* (Pyojun Korean Dictionary; Seoul: National Institute of Korean Language, 2019). Sixteen item sets were constructed as experimental stimuli. An example of the stimuli with four conditions is presented in Table 1.

**TABLE 1:** Example items in the four conditions of the experiment.

Region 1	Region 2	Region 3	Region 4	Region 5	Region 6	Region 7
**A. Adjective; categorially ambiguous**						
Minsu-neun	yeog-eseo	jeongmal	***neuj-eun***	chingu-leul	neugeusi	gidaly-eoss-da.
Minsu-TOP	station-LOC	really	late-PRS.ADN	friend-ACC	patiently	wait-PST-DECL
‘Minsu waited patiently at the station for his friend who was really late.’						
**B. Verb; categorially ambiguous**						
Minsu-neun	yeog-eseo	jeongmal	***neuj-neun***	chingu-leul	neugeusi	gidaly-eoss-da.
Minsu-TOP	station-LOC	really	become.late-PRS.ADN	friend-ACC	patiently	wait-PST-DECL
‘Minsu waited patiently at the station for his friend who was really becoming late.’						
**C. Adjective; categorially unambiguous**						
Minsu-neun	yeog-eseo	jeongmal	***manh-eun***	chingu-leul	neugeusi	gidaly-eoss-da.
Minsu-TOP	station-LOC	really	many-PRS.ADN	friend-ACC	patiently	wait-PST-DECL
‘Minsu waited patiently at the station for his really many friends.’						
**D. Verb; categorially unambiguous**						
Minsu-neun	yeog-eseo	jeongmal	***o-neun***	chingu-leul	neugeusi	gidaly-eoss-da.
Minsu-TOP	station-LOC	really	come-PRS.ADN	friend-ACC	patiently	wait-PST-DECL
‘Minsu waited patiently at the station for his friend who was indeed coming.’						

Conditions A and B contain categorially ambiguous words, with condition A presenting the base adjectives and condition B presenting the adjective-derived verbs. Conditions C and D serve as the baseline conditions, where condition C presents the categorially unambiguous adjectives and condition D presents the categorially unambiguous verbs. The region with bold lettering (Region 4) represents the Critical Region (categorially (un)ambiguous adjectives and verbs in Korean). The regions following the critical region (Region 4) represent the spillover region (Region 5) and second spillover region (Region 6), respectively.

In addition to the critical experimental items,^3^ we also included 36 filler items that were not pertinent to the present experimental manipulation.

Prior to the real-time processing experiment, we conducted an acceptability rating experiment (31 native speakers of Korean volunteered to participate in the experiment) to ensure that the processing difficulty manifested in longer reading times in our experiment would not be due to the unnaturalness of the inflectional structures used in our stimuli or to semantic anomaly. We expected that all conditions would be rated acceptable. The norming study revealed that all conditions were rated above 3.5 out of 7, suggesting that the acceptability of all sentences was above average.

The experiment was conducted on PC IbexFarm, which is a web-based demonstration platform (Zehr and Schwarz, 2018). All stimuli were pseudo-randomized in a Latin-square to ensure that the same condition in the identical item would not occur consecutively. Through the experimental link generated by PC IbexFarm, participants completed the experiment on their own laptops. Participants were instructed to press the space bar to read the sentences word-by-word at their own speed. After each sentence, there was a comprehension question (e.g., “*Did somebody see anything?*”), and participants were instructed to press *ye* ‘yes’ or *aniyo* ‘no’ to indicate their judgement.^4^

### 3.3 Prediction

If these words are listed under separate lexical entries (Koo, 2010; Yang, 2015; for English, see Jackendoff, 1976; Lieber, 1980; ao), we expect greater processing costs for the verbs (conditions B and D) than adjectives (conditions A and C) in general, regardless of whether the word is categorially ambiguous or not, owing to the larger size of inflectional forms on verbs than on adjectives (cf. Traficante and Burani, 2003; also see Sereno and Jongman, 1997 for the greater processing difficulty in verbs than nouns).

Under the separate-entry account (e.g., Koo, 2010; Yang, 2015), another possibility is that we may see greater processing difficulty for categorially ambiguous words (conditions A and B) compared to unambiguous ones (conditions C and D) due to competition in word-activation (interactive activation model; Reicher, 1969; McClelland and Rumelhart, 1981; Rumelhart and McClelland, 1982). Under either possibility, we do not expect an interaction between *Category* and *Category Ambiguity*.

In contrast, following a theory that predicts that these words involve a process of zero derivation (Song, 1988; Koo, 2021; for English, see Marchand, 1964; Myers, 1984; Lukic, 2016; Krasuska and Yoshida, 2019; Lukic et al., 2023), the processing difficulty should be associated with word-internal structure building. Thus, we expect that adjective-derived verbs (condition B) would induce greater processing difficulty than the base adjectives (condition A) due to the morphological complexity and additional step in adding a covert category-changing morpheme in real-time processing. However, such morphological complexity effect should not be seen in categorially unambiguous verbs (condition D) compared to unambiguous adjectives (condition C), as they should be equally less complex in word-internal structure. In other words, we expect an interaction between *Category* and *Category Ambiguity*.

### 3.4 Analysis and results

**FIGURE 1:**
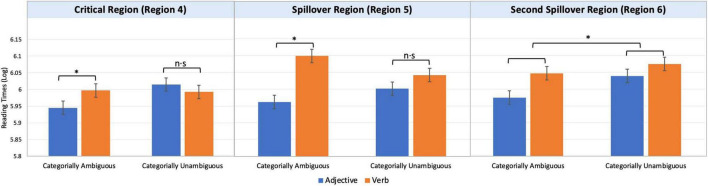
Mean reading times at the critical region (region 4), spillover region (region 5), and second spillover region (region 6) for the four conditions. Asterisks (*) indicate significant differences across conditions (‘*’ indicates *p* < 0.05, and ‘n.s’ indicates non-significant).

The results were analyzed with a linear mixed effect regression model using the lme4 package (Bates et al., 2015) with fixed effects for *Category* (adjective vs. verb) and *Category Ambiguity* (categorially ambiguous vs. categorially unambiguous) and random effects for both participants and experimental items (Baayen et al., 2008).^5^ Each model for individual region included the maximal random effect structure provided the model converged. Reading times for the critical region (Region 4), spillover region (Region 5), and second spillover region (Region 6) were log-transformed with the purpose of minimizing non-normality (Box and Cox, 1964). Figure 1 shows the mean reading times at the critical region (Region 4), spillover region (Region 5), and second spillover region (Region 6) for the four conditions.

We began by focusing on the critical region (Region 4). We found an interaction between *Category* and *Category Ambiguity* (β = 0.07, SE = 0.04, *t* = 2.03). Crucially, the planned comparisons showed a significant simple effect of *Category* within the categorially ambiguous adjective/verb conditions (conditions A and B) such that condition A was read faster than condition B (β = 0.05, SE = 0.03, *t* = 1.98). However, the same effect was absent in the categorially unambiguous adjective/verb conditions (conditions C and D); the reading times for condition C did not differ from those for condition D (β = −0.02, SE = 0.03, *t* = −0.87). We found no main effect of *Category* (β = 0.01, SE = 0.02, *t* = 0.81) or *Category Ambiguity* (β = −0.03, SE = 0.02, *t* = −1.79).

The interaction between *Category* and *Category Ambiguity* persisted into the spillover region (Region 5: β = 0.10, SE = 0.04, *t* = 2.20). Crucially, the planned comparisons showed a significant simple effect of *Category* within the categorially ambiguous adjective/verb conditions (conditions A and B), such that condition A was read faster than condition B (β = 0.14, SE = 0.03, *t* = 4.11). However, the same effect was absent in the categorially unambiguous adjective/verb conditions (conditions C and D); the reading times for condition C did not differ from that for condition D (β = 0.05, SE = 0.03, *t* = 1.59). Additionally, a significant main effect of *Category* was observed, such that the reading times for the adjectives (conditions A and C) were shorter than for the verbs (conditions B and D) (β = 0.09, SE = 0.02, *t* = 4.20). We found no effect of *Category Ambiguity* (β = 0.01, SE = 0.02, *t* = 0.41).

At the second spillover region (Region 6), we found a main effect of *Category* (β = 0.06, SE = 0.02, *t* = 2.99) such that adjectives (conditions A and C) were read faster than verbs (conditions B and D), and *Category Ambiguity* (β = −0.05, SE = 0.02, *t* = −2.36)^6^ such that categorially unambiguous adjective/verb sentences (conditions C and D) were read slower than the categorially ambiguous ones (conditions A and B). However, there was no interaction between these two (β = 0.04, SE = 0.04, *t* = 0.97).^7^

Table 2 illustrates the statistical by-region (regions 4, 5, and 6) analyses of the linear mixed model for the experiment.

**TABLE 2:** Statistical analyses of the linear mixed model by the critical region (Region 4), spillover region (Region 5), and second spillover region (Region 6) from the self-paced Reading experiment.

	Estimate	SE	*t*-value	*p*-value
**Critical region (Region 4)**				
(Intercept)	6.00	0.05	130.03	
*Category*	0.01	0.02	0.81	0.42
*Category Ambiguity*	−0.03	0.02	−1.79	0.08
***Category*** × ***Category Ambiguity***	**0.07**	**0.04**	**2.03**	0.04*
**Spillover region (Region 5)**				
(Intercept)	6.04	0.05	121.05	
***Category***	**0.09**	**0.02**	**4.20**	3.326e-05***
*Category Ambiguity*	0.01	0.02	0.41	0.68
***Category*** × ***Category Ambiguity***	**0.10**	**0.04**	**2.20**	0.03*
**Second spillover region (Region 6)**				
(Intercept)	6.05	0.05	122.87	
***Category***	**0.06**	**0.02**	**2.99**	0.00**
***Category Ambiguity***	**−0.05**	**0.02**	**−2.36**	0.02*
*Category* × *Category Ambiguity*	0.04	0.04	0.97	0.33

Each model included simple difference sum-coded fixed effects of *Category* (adjectives being coded as −0.5; verbs being coded as 0.5) and *Category Ambiguity* (categorially ambiguous being coded as 0.5; unambiguous being coded as −0.5) of the words. Asterisks (*) indicate significant differences across conditions (“*” indicates *p* < 0.05, “**” indicates *p* < 0.01, “***” indicates *p* < 0.001). Bold values indicate the significant effects observed in our experiment.

## 4 Discussion

This study investigated the processing of categorially ambiguous adjectives and verbs in Korean, through which we provided new insights into the theories of the lexicon. Following a theory that suggests these words be listed separately (e.g., Lieber, 1980; Koo, 2010; Yang, 2015), we should see greater processing difficulty in one class than the other (e.g., Sereno and Jongman, 1997; Tyler et al., 2001; Traficante and Burani, 2003) or in categorially ambiguous words than unambiguous ones in general. Word processing can be competitive as the activation of a word occurs simultaneously at several levels, and activating a word would spread activation to a phonologically and semantically similar word (interactive activation model; Reicher, 1969; McClelland and Rumelhart, 1981; Rumelhart and McClelland, 1982). Consequently, the activation of a categorially ambiguous word would be impeded, as the activation of one ([_ADJ_ *neujda* ‘late’]) competes with the other ([_V_ *neujda* ‘become late’]) or vice versa, due to being phonologically identical and semantically related.

Following a theory that suggests that these words share one single lexical entry, with one class being morphologically derived from the other (e.g., Marchand, 1964; Myers, 1984; Song, 1988; Koo, 2021), we should see increased processing difficulty for the derived form but not for the base form (Lukic, 2016; Krasuska and Yoshida, 2019; Lukic et al., 2023). Upon encountering a categorially ambiguous word, the reader builds the structure for the base form, attaches a zero morpheme to achieve the derived-verb form, and then attaches the inflectional suffix to integrate the inflected verb into the sentence structure (e.g., [_ADN_ [_V_ [_ADJ_ *neuj* ‘late’]-Ø ‘become’]-*nuen*]). The additional derivation step in the structure building for adjective-derived verbs would induce greater costs than base adjectives and categorially unambiguous words, because base adjectives and categorially unambiguous words can be integrated into the sentence by adding the inflectional suffix only (e.g., base adjective: [_ADN_ [_ADJ_ *neuj* ‘late’]-*uen*]; categorially unambiguous adjective and verb: [_ADN_ [_ADJ_ *manh* ‘many’]-*uen*], [_ADN_ [_V_ *o* ‘come’]-*nuen*], respectively).

In this study, adjective-derived verbs were read slower than the base adjectives at the critical and spillover regions. However, such an effect was not found between categorially unambiguous verbs and adjectives. This suggests that readers recognized the base category and built the word-internal structure during real-time sentence processing.

Our study also aligned with what Lukic et al. (2023) observed: readers were sensitive to the morphological complexity brought by the covert element. Crucially, by focusing on one derivational direction (adjective-derived verb), we were able to compare the processing differences between categorially (un)ambiguous adjectives and (adjective-derived) verbs directly. In particular, though Korean adjectives and verbs undergo inflections, similar to English nouns and verbs, they differ in that morphosyntactic cues (e.g., -*eun* indicates relativized adjectives; -*neun* indicates relativized verbs) play an essential role in distinguishing the word category. The absence of greater processing difficulty in categorially unambiguous verbs versus unambiguous adjectives (compared to that observed in adjective-derived verbs vs. their base adjective counterparts) suggests that processing difficulty is associated with the covert structure (Lukic, 2016; Krasuska and Yoshida, 2019; Lukic et al., 2023).

We controlled for the word length and semantic plausibility by conducting an acceptability rating test prior to our real-time experiment. However, related processing factors such as argument structure information were not controlled, as the categorially ambiguous adjectives and verbs investigated in our study were stative predicates. Future studies could be conducted in the other direction (i.e., verb-derived adjective) with the control of other independent factors to examine whether processing difficulty would occur differently.

## 5 Conclusion

This study provides experimental evidence of the effect of covert structure on categorially ambiguous adjectives and verbs in Korean. By conducting a self-paced reading experiment, we observed reading time slowdown in adjective-derived verbs compared to their base adjective counterparts but not in categorially unambiguous conditions. This suggests that readers are sensitive to the covert structure. This study adds crosslinguistic evidence of zero derivation in categorially ambiguous words, whose results offer new insight into the theories of the lexicon (Marchand, 1964; Jackendoff, 1976; Clark and Clark, 1979; Lieber, 1980; Myers, 1984; Song, 1988; Don, 2005; Koo, 2010, 2021; Yang, 2015; Lukic, 2016; Krasuska and Yoshida, 2019; Lukic et al., 2023).

## Data availability statement

The original contributions presented in this study are included in this article/Supplementary material, further inquiries can be directed to the corresponding author.

## Author contributions

NK, ZL, SB, and CL conceived the study. SB, CL, and ZL created the stimuli. NK supervised the stimuli creation and conducted statistical analyses of the data. NK and ZL implemented the experiment. All authors contributed to planning the research, participated in writing this article, and approved the submitted version.

## Footnotes

1 In Korean, adjectives and verbs can be distinguished by their morphosyntactic inflectional forms; for example, [_ADJ_ *balgda* ‘bright’] does not need any overt suffix in forming a present tense declarative, as in (1a), whereas for [_V_ *balgda* ‘become bright’], the overt suffix -*neun* must be attached to the verb stem to form the present tense declarative, as in (1b) (Sohn, 1999, 2004; Song, 2014; Lee et al., 2015). This study does not argue whether Korean has the category of adjectives (ADJ), which we take for granted. For arguments on this issue, see Kim (2002, 2007), Sohn (2004), Choe (2005), Kang (2005).

2 In the Korean verb phrase *meog-go.iss-eossda* (‘eat’-‘ing’-past) ‘was eating,’ *-go.issda* expresses the progressive aspect, and *-eossda* expresses the past tense (Sohn, 1999). In English, the suffix *-ing* in *singing* can mark progressive, as in “John is singing a song,” or form nominalization as in “John’s singing of the song” (Bauke and Roeper, 2012). The morpheme *-s* in *sings* can also express third-person singular and present tense.

3 Ideally, the categorially ambiguous pair should have similar word frequency in both the adjective and the verb category (cf. Brysbaert et al., 2018). However, we could not control for frequency owing to several factors. First, there is a very limited number of categorially ambiguous adjectives and (adjective-derived) verbs in Korean (Koo, 2021). Second, we used the adnominal form (e.g., [_ADN_ [_ADJ_ *neuj*]-*eun*]; [_ADN_ [_V_ *neuj*]-*neun*]) and the indicative form (e.g., [_IND_ [_ADJ_ *keu*]-*da*]; [_IND_ [_V_ *keun*]-*da*]) to distinguish the adjectives from verbs as well as to control for the word length (both forms contained two syllables). Both the adnominal and indicative forms that provide strong cues for either the adjective or the verb tend to appear more frequently with adjectives than verbs in general, according to the *Sejong Corpus* (Kang and Kim, 2004). Consequently, the processing differences between conditions A and B may not be due solely to the frequency itself. To confirm how strongly the frequency may affect the processing of the categorially ambiguous adjective/verb pairs, we conducted a follow-up study. This is reported in footnote 7.

4 Participants were given immediate feedback after each question, and the comprehension accuracy was 93%.

5 lmer(RT ∼ *Category***CategoryAmbiguity* + (1| Subj) + (1| ItemCode), REML = FALSE, data = data, control = lmerControl(optimizer = “bobyqa”))

6 Unexpectedly, the categorially unambiguous adjectives/verbs were read slower than the categorially ambiguous ones. We speculated that this might reflect some semantic interference effects and leave this for a future study.

7 As the words were not balanced for frequency in conditions A and B for the reasons stated in footnote 3, we conducted a follow-up experiment to further test whether the processing differences between conditions A and B, which were not found between conditions C and D, can be attributed to the covert structure-building process involved in zero-derivation and not to mere lexical frequency. The stimuli for conditions A and B remained intact, but the word frequency for the adjectives in condition C was designed to be overwhelmingly higher than the verbs in condition D based on the *Sejong Corpus* (Kang and Kim, 2004). We continued to find a significant simple effect of *Category* only within the categorially ambiguous adjectives/verbs at the critical region (β = 0.08, SE = 0.04, *t* = 2.18) and the spillover region (β = 0.10, SE = 0.04, *t* = 2.28), suggesting that the reading time slowdown in the categorially ambiguous verbs may not be solely due to the higher frequency of adjectives than verbs. We direct readers to the Supplementary material for more details regarding the follow-up experiment.
