# Supplementary material for: Zero-derivation in Korean: the effect of covert structure in real-time processing
Source: Front Psychol. 2023 Dec 13;14:1230927. doi: 10.3389/fpsyg.2023.1230927 (PMC10751375; doi:10.3389/fpsyg.2023.1230927)
Supplement: Supplementary file 1 [file Data_Sheet_1.docx]

Supplementary Material

Zero-derivation in Korean: The Effect of Covert Structure in Real-time Processing

Nayoun Kim*, Ziying Li, Seonghyeon Byeon, Chaejin Lee

***Correspondence:** Nayoun Kim: [nayoun@skku.edu](mailto:nayoun@skku.edu)

# Sample stimuli used in the acceptability rating and self-paced reading experiments

Condition A: Adjective; Categorially Ambiguous

Condition B: Verb; Categorially Ambiguous

Condition C: Adjective; Categorially Unambiguous

Condition D: Verb; Categorially Unambiguous

***Note***. Conditions A and B contain categorially ambiguous words, with Condition A presenting the base adjective and Condition B presenting the adjective-derived verb. Conditions C and D serve as the baseline conditions, where Condition C presents the categorially unambiguous adjectives and Condition D presents the categorially unambiguous verbs. Regions 4, 5, and 6 represent the critical region, spillover region, and second spillover region, respectively, and the region in bold letter represents the critical region (categorially (un)ambiguous adjectives and verbs in Korean; Region 4).

|  | Region 1 | Region 2 | Region 3 | Region 4  (critical region) | Region 5  (spillover region) | Region 6  (spillover region 2) | Region 7 |
| --- | --- | --- | --- | --- | --- | --- | --- |
| **ITEM 1** | **A. Adjective; Categorially Ambiguous** | | | | | | |
|  | 도희는 | 진돗개가 | 최근에 | **크다** | 여겨서 | 간식을 | 먹였다. |
|  | Dohui-neun | jindosgae-ga | choegeun-e | **keu-da** | yeogyeoseo | gansig-eul | meog-yeoss-da |
|  | Dohui-TOP | Jindo.dog-NOM | recently-AD | big-PRS.IND | think-CAUSE | snack-ACC | feed-PST-DECL |
|  | “Dohui thought the Jindo dog was big recently, so she fed it snacks.” | | | | | | |
|  | 1. **B. Verb; Categorially Ambiguous** | | | | | | |
|  | 도희는 | 진돗개가 | 최근에 | **큰다** | 여겨서 | 간식을 | 먹였다. |
|  | Dohui-neun | jindosgae-ga | choegeun-e | **keun-da** | yeogyeoseo | gansig-eul | meog-yeoss-da |
|  | Dohui-TOP | Jindo.dog-NOM | recently-AD | become.big-PRS.IND | think-CAUSE | snack-ACC | feed-PST-DECL |
|  | “Dohui thought the Jindo dog was becoming big recently, so she fed it snacks.” | | | | | | |
|  | 1. **C. Adjective; Categorially Unambiguous** | | | | | | |
|  | 도희는 | 진돗개가 | 최근에 | **작다** | 여겨서 | 간식을 | 먹였다. |
|  | Dohui-neun | jindosgae-ga | choegeun-e | **jag-da** | yeogyeoseo | gansig-eul | meog-yeoss-da |
|  | Dohui-TOP | Jindo.dog-NOM | recently-AD | small-PRS.IND | think-CAUSE | snack-ACC | feed-PST-DECL |
|  | “Dohui thought the Jindo dog was small recently, so she fed it snacks.” | | | | | | |
|  | 1. **D. Verb; Categorially Unambiguous** | | | | | | |
|  | 도희는 | 진돗개가 | 최근에 | **뛴다** | 여겨서 | 간식을 | 먹였다. |
|  | Dohui-neun | jindosgae-ga | choegeun-e | **ttwin-da** | yeogyeoseo | gansig-eul | meog-yeoss-da |
|  | Dohui-TOP | Jindo.dog-NOM | recently-AD | run-PRS.IND | think-CAUSE | snack-ACC | feed-PST-DECL |
|  | “Dohui thought the Jindo dog had been running recently, so she fed it snacks.” | | | | | | |

|  | Region 1 | Region 2 | Region 3 | Region 4  (critical region) | Region 5  (spillover region) | Region 6  (spillover region 2) | Region 7 | Region 8 |
| --- | --- | --- | --- | --- | --- | --- | --- | --- |
| **ITEM 2** | 1. **A. Adjective; Categorially Ambiguous** | | | | | | | |
|  | 준기는 | 예상보다 | 더 | **늦은** | 회의에 | 곧바로 | 집으로 | 갔다. |
|  | Jungi-neun | yesang-boda | deo | **neuj-eun** | hoeui-e | godbalo | jib-eulo | ga-ss-da |
|  | Jungi-TOP | expect-AD | more | late-PRS.ADN | meeting-CAUSE | right.away | home-AD | go-PST-DECL |
|  | “Jungi went straight home after a meeting that was later than expected.” | | | | | | | |
|  | 1. **B. Verb; Categorially Ambiguous** | | | | | | | |
|  | 준기는 | 예상보다 | 더 | **늦는** | 회의에 | 곧바로 | 집으로 | 갔다. |
|  | Jungi-neun | yesang-boda | deo | **neuj-neun** | hoeui-e | godbalo | jib-eulo | ga-ss-da |
|  | Jungi-TOP | expect-AD | more | become.late-PRS.ADN | meeting-CAUSE | right.away | home-AD | go-PST-DECL |
|  | “Jungi went straight home after a meeting a meeting that ran later than expected.” | | | | | | | |
|  | 1. **C. Adjective; Categorially Unambiguous** | | | | | | | |
|  | 준기는 | 예상보다 | 더 | **이른** | 회의에 | 곧바로 | 집으로 | 갔다. |
|  | Jungi-neun | yesang-boda | deo | **il-eun** | hoeui-e | godbalo | jib-eulo | ga-ss-da |
|  | Jungi-TOP | expect-AD | more | be.early-PRS.ADN | meeting-CAUSE | right.away | home-AD | go-PST-DECL |
|  | “Jungi went straight home after a meeting that was earlier than expected.” | | | | | | | |
|  | 1. **D. Verb; Categorially Unambiguous** | | | | | | | |
|  | 준기는 | 예상보다 | 더 | **걸린** | 회의에 | 곧바로 | 집으로 | 갔다. |
|  | Jungi-neun | yesang-boda | deo | **geol-lin** | hoeui-e | godbalo | jib-eulo | ga-ss-da |
|  | Jungi-TOP | expect-AD | more | take.time-PRS.ADN | meeting-CAUSE | right.away | home-AD | go-PST-DECL |
|  | “Jungi went home right away because of a meeting that took longer than expected.” | | | | | | | |

|  | Region 1 | Region 2 | Region 3 | Region 4  (critical region) | Region 5  (spillover region) | Region 6  (spillover region 2) | Region 7 |
| --- | --- | --- | --- | --- | --- | --- | --- |
| **ITEM 3** | 1. **A. Adjective; Categorially Ambiguous** | | | | | | |
|  | 동별이는 | 지금 | 엄청 | **밝은** | 날이라 | 창문을 | 열었다. |
|  | Dongbyeoli-neun | jigeum | eomcheong | **balg-eun** | nal-ila | changmun-eul | yeol-eoss-da |
|  | Dongbyeoli-TOP | now | much | bright-PRS.ADN | day-BECAUSE | window-ACC | open-PST-DECL |
|  | “Dongbyeol opened the window because it was a very bright day.” | | | | | | |
|  | 1. **B. Verb; Categorially Ambiguous** | | | | | | |
|  | 동별이는 | 지금 | 엄청 | **밝는** | 날이라 | 창문을 | 열었다. |
|  | Dongbyeoli-neun | jigeum | eomcheong | **balg-neun** | nal-ila | changmun-eul | yeol-eoss-da |
|  | Dongbyeoli-TOP | now | much | dawn-PRS.ADN | day-BECAUSE | window-ACC | open-PST-DECL |
|  | “Dongbyeol opened the window because it was a very dawning day.” | | | | | | |
|  | 1. **C. Adjective; Categorially Unambiguous** | | | | | | |
|  | 동별이는 | 지금 | 엄청 | **더운** | 날이라 | 창문을 | 열었다. |
|  | Dongbyeoli-neun | jigeum | eomcheong | **deo-un** | nal-ila | changmun-eul | yeol-eoss-da |
|  | Dongbyeoli-TOP | now | much | hot-PRS.ADN | day-BECAUSE | window-ACC | open-PST-DECL |
|  | “Dongbyeol opened the window because it was a very hot day.” | | | | | | |
|  | 1. **D. Verb; Categorially Unambiguous** | | | | | | |
|  | 동별이는 | 지금 | 엄청 | **끓는** | 날이라 | 창문을 | 열었다. |
|  | Dongbyeoli-neun | jigeum | eomcheong | **kkeulh-neun** | nal-ila | changmun-eul | yeol-eoss-da |
|  | Dongbyeoli-TOP | now | much | boil-PRS.ADN | day-BECAUSE | window-ACC | open-PST-DECL |
|  | “Dongbyeol opened the window because it was a boiling day.” | | | | | | |

|  | Region 1 | Region 2 | Region 3 | Region 4  (critical region) | Region 5  (spillover region) | Region 6  (spillover region 2) | Region 7 |
| --- | --- | --- | --- | --- | --- | --- | --- |
| **ITEM 4** | 1. **A. Adjective; Categorially Ambiguous** | | | | | | |
|  | 승민이는 | 아이가 | 특히 | **크다** | 들어서 | 놀라고 | 말았다. |
|  | Seungmini-neun | ai-ga | teughi | **keu-da** | deuleo-seo | nolla-go | mal-ass-da |
|  | Seungmini-TOP | child-NOM | especially | big-PRS.ADN | hear-CAUSE | surprise-LNK | end.up-PST-DECL |
|  | “Seungmin was surprised to hear that the child was especially big.” | | | | | | |
|  | 1. **B. Verb; Categorially Ambiguous** | | | | | | |
|  | 승민이는 | 아이가 | 특히 | **큰다** | 들어서 | 놀라고 | 말았다. |
|  | Seungmini-neun | ai-ga | teughi | **keu-n-da** | deuleo-seo | nolla-go | mal-ass-da |
|  | Seungmini-TOP | child-NOM | especially | grow-PRS-ADN | hear-CAUSE | surprise-LNK | end.up-PST-DECL |
|  | “Seungmin was surprised to hear that the child was growing up particularly fast.” | | | | | | |
|  | 1. **C. Adjective; Categorially Unambiguous** | | | | | | |
|  | 승민이는 | 아이가 | 특히 | **작다** | 들어서 | 놀라고 | 말았다. |
|  | Seungmini-neun | ai-ga | teughi | **jag-da** | deuleo-seo | nolla-go | mal-ass-da |
|  | Seungmini-TOP | child-NOM | especially | small-PRS.ADN | hear-CAUSE | surprise-LNK | end.up-PST-DECL |
|  | “Seungmin was surprised to hear that the child was especially small.” | | | | | | |
|  | 1. **D. Verb; Categorially Unambiguous** | | | | | | |
|  | 승민이는 | 아이가 | 특히 | **잔다** | 들어서 | 놀라고 | 말았다. |
|  | Seungmini-neun | ai-ga | teughi | **jan-da** | deuleo-seo | nolla-go | mal-ass-da |
|  | Seungmini-TOP | child-NOM | especially | sleep-PRS.IND | hear-CAUSE | surprise-LNK | end.up-PST-DECL |
|  | “Seungmin was surprised to hear that the child was sleeping particularly well.” | | | | | | |

|  | Region 1 | Region 2 | Region 3 | Region 4  (critical region) | Region 5  (spillover region) | Region 6  (spillover region 2) | Region 7 |
| --- | --- | --- | --- | --- | --- | --- | --- |
| **ITEM 5** | 1. **A. Adjective; Categorially Ambiguous** | | | | | | |
|  | 민수는 | 역에서 | 정말 | **늦은** | 친구를 | 느긋이 | 기다렸다. |
|  | Minsu-neun | yeog-eseo | jeongmal | **neuj-eun** | chingu-leul | neugeusi | gidaly-eoss-da. |
|  | Minsu-TOP | station-LOC | really | late-PRS.ADN | friend-ACC | patiently | wait-PST-DECL |
|  | “Minsu waited patiently at the station for his friend who was really late.” | | | | | | |
|  | 1. **B. Verb; Categorially Ambiguous** | | | | | | |
|  | 민수는 | 역에서 | 정말 | **늦는** | 친구를 | 느긋이 | 기다렸다. |
|  | Minsu-neun | yeog-eseo | jeongmal | **neuj-neun** | chingu-leul | neugeusi | gidaly-eoss-da. |
|  | Minsu-TOP | station-LOC | really | become.late-PRS.ADN | friend-ACC | patiently | wait-PST-DECL |
|  | “Minsu waited patiently at the station for his friend who was really becoming late.” | | | | | | |
|  | 1. **C. Adjective; Categorially Unambiguous** | | | | | | |
|  | 민수는 | 역에서 | 정말 | **많은** | 친구를 | 느긋이 | 기다렸다. |
|  | Minsu-neun | yeog-eseo | jeongmal | **manh-eun** | chingu-leul | neugeusi | gidaly-eoss-da. |
|  | Minsu-TOP | station-LOC | really | many-PRS.ADN | friend-ACC | patiently | wait-PST-DECL |
|  | “Minsu waited patiently at the station for his really many friends.” | | | | | | |
|  | 1. **D. Verb; Categorially Unambiguous** | | | | | | |
|  | 민수는 | 역에서 | 정말 | **오는** | 친구를 | 느긋이 | 기다렸다. |
|  | Minsu-neun | yeog-eseo | jeongmal | **o-neun** | chingu-leul | neugeusi | gidaly-eoss-da. |
|  | Minsu-TOP | station-LOC | really | come-PRS.ADN | friend-ACC | patiently | wait-PST-DECL |
|  | “Minsu waited patiently at the station for his friend who was indeed coming.” | | | | | | |

**2. Results of the acceptability rating experiment**

**Table 1**. **Fixed effects of the linear mixed effect model from the acceptability rating experiment.**

|  | Estimate | SE | t-value |
| --- | --- | --- | --- |
| (Intercept) | 4.3517 | 0.2462 | 17.677 |
| Category | −1.1700 | 0.2874 | −4.071 |
| Category Ambiguity | −0.4877 | 0.1718 | −2.839 |
| Category **×** Category Ambiguity | −0.3894 | 0.4292 | −0.907 |

**Figure 1. The mean ratings and standard error for four conditions in the acceptability rating experiment.**


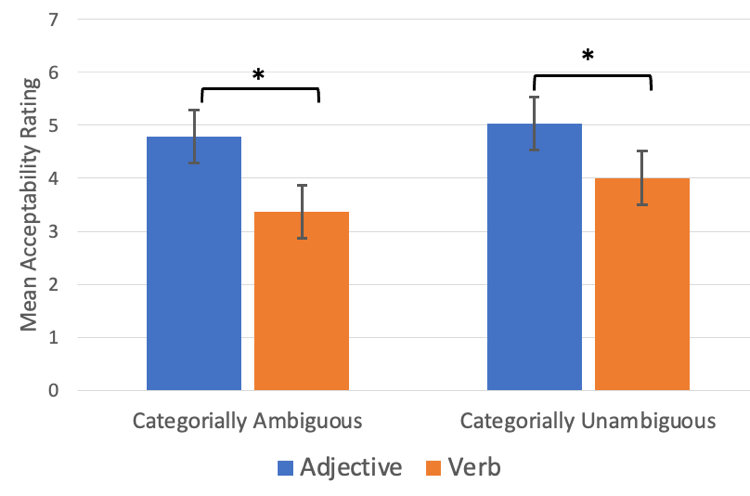


***Note*.** Asterisks (*) indicate significant differences across conditions (‘*’ indicates p<0.05 and ‘n·s’ indicates non-significant).

**3. Sample stimuli used in the follow-up self-paced reading experiment**

Condition A: Adjective, Categorially Ambiguous

Condition B: Verb, Categorially Ambiguous

Condition C: Adjective, Categorially Unambiguous

Condition D: Verb, Categorially Unambiguous

***Note***. Conditions A and B contain categorially ambiguous words, with Condition A presenting the base adjective and Condition B presenting the adjective-derived verb. Conditions C and D serve as the baseline conditions, where Condition C presents the categorially unambiguous adjectives and Condition D presents the categorially unambiguous verbs. Regions 4, 5, and 6 represent the critical region, spillover region, and second spillover region, respectively, and the region in bold letter represents the critical region (categorially (un)ambiguous adjectives and verbs in Korean; Region 4).

|  | Region 1 | Region 2 | Region 3 | Region 4  (critical region) | Region 5  (spillover region) | Region 6  (spillover region 2) | Region 7 | Region 8 |
| --- | --- | --- | --- | --- | --- | --- | --- | --- |
| **ITEM 1** | 1. **A. Adjective; Categorially Ambiguous** | | | | | | | |
|  | 동혁이는 | 밭에서 | 아주 | **붉은** | 고추를 | 따다가 | 씻어서 | 먹었다. |
|  | Donghyeogi-neun | bat-eseo | aju | **bulg-eun** | gochu-reul | ttadaga | ssis-eoseo | meog-ess-da. |
|  | Donghyeog-Top | field-LOC | very | red-PRS.ADN | pepper-ACC | pick-AFTER | wash-AFTER | eat-PST-DECL |
|  | “Donghyuk picked a pepper which was very red in the field, washed, and ate it.” | | | | | | | |
|  | 1. **B. Verb; Categorially Ambiguous** | | | | | | | |
|  | 동혁이는 | 밭에서 | 아주 | **붉는** | 고추를 | 따다가 | 씻어서 | 먹었다. |
|  | Donghyeogi-neun | bat-eseo | aju | **bung-neun** | gochu-reul | ttadaga | ssis-eoseo | meog-ess-da. |
|  | Donghyeog-Top | field-LOC | very | become.red-PRS.ADN | pepper-ACC | pick-AFTER | wash-AFTER | eat-PST-DECL |
|  | “Donghyuk picked a red pepper which was becoming very red in the field, washed, and ate it.” | | | | | | | |
|  | 1. **C. Adjective; Categorially Unambiguous** | | | | | | | |
|  | 동혁이는 | 밭에서 | 아주 | **작은** | 고추를 | 따다가 | 씻어서 | 먹었다. |
|  | Donghyeogi-neun | bat-eseo | aju | **jag-eun** | gochu-reul | ttadaga | ssis-eoseo | meog-ess-da. |
|  | Donghyeog-Top | field-LOC | very | small-PRS.ADN | pepper-ACC | pick-AFTER | wash-AFTER | eat-PST-DECL |
|  | “Donghyuk picked a red pepper which was very small in the field, washed, and ate it.” | | | | | | | |
|  | 1. **D. Verb; Categorially Unambiguous** | | | | | | | |
|  | 동혁이는 | 밭에서 | 아주 | **익는** | 고추를 | 따다가 | 씻어서 | 먹었다. |
|  | Donghyeogi-neun | bat-eseo | aju | **ig-neun** | gochu-reul | tadaga | ssis-eoseo | meog-ess-da. |
|  | Donghyeog-Top | field-LOC | very | become.ripe-PRS.ADN | pepper-ACC | pick-AFTER | wash-AFTER | eat-PST-DECL |
|  | “Donghyuk picked a red pepper which is very ripped in the field, washed, and ate it.” | | | | | | | |

|  | Region 1 | Region 2 | Region 3 | Region 4  (critical region) | Region 5  (spillover region) | Region 6  (spillover region 2) | Region 7 | Region 8 |
| --- | --- | --- | --- | --- | --- | --- | --- | --- |
| **ITEM 2** | 1. **A. Adjective; Categorially Ambiguous** | | | | | | | |
|  | 우준이는 | 부질없이 | 항상 | **밝은** | 날이면 | 밖으로 | 나가서 | 놀았다. |
|  | Ujuni-neun | bujireopsi | hangsang | **balg-eun** | nal-imyeon | bakkeuro | naga-seo | nol-ass-da. |
|  | Ujun-Top | without.any. fuss | always | bright-PRS.ADN | day-IF | out-LOC | go-AFTER | play-PST-DECL |
|  | “Ujun always went out and played on bright days without any fuss.” | | | | | | | |
|  | 1. **B. Verb; Categorially Ambiguous** | | | | | | | |
|  | 우준이는 | 부질없이 | 항상 | **밝는** | 날이면 | 밖으로 | 나가서 | 놀았다. |
|  | Ujuni-neun | bujireopsi | hangsang | **balg-neun** | nal-imyeon | bakk-euro | naga-seo | nol-ass-da. |
|  | Ujun-Top | without.any. fuss | always | become.bright-PRS.ADN | day-IF | out-LOC | go-AFTER | play-PST-DECL |
|  | “Ujun always went out and played on brightening days without any fuss.” | | | | | | | |
|  | 1. **C. Adjective; Categorially Unambiguous** | | | | | | | |
|  | 우준이는 | 부질없이 | 항상 | **좋은** | 날이면 | 밖으로 | 나가서 | 놀았다. |
|  | Ujuni-neun | bujireopsi | hangsang | **jo-eun** | nal-imyeon | bakk-euro | naga-seo | nol-ass-da. |
|  | Ujun-Top | without.any. fuss | always | nice-PRS.ADN | day-IF | out-LOC | go-AFTER | play-PST-DECL |
|  | “Ujun always went out and played on nice days without any fuss.” | | | | | | | |
|  | 1. **D. Verb; Categorially Unambiguous** | | | | | | | |
|  | 우준이는 | 부질없이 | 항상 | **쉬는** | 날이면 | 밖으로 | 나가서 | 놀았다. |
|  | Ujuni-neun | bujireopsi | hangsang | **swi-neun** | nal-imyeon | bakk-euro | naga-seo | nol-ass-da. |
|  | Ujun-Top | without.any. fuss | always | rest-PRS.ADN | day-IF | out-LOC | go-AFTER | play-PST-DECL |
|  | “Ujun always went out and played on resting days without any fuss.” | | | | | | | |

|  | Region 1 | Region 2 | Region 3 | Region 4  (critical region) | Region 5  (spillover region) | Region 6  (spillover region 2) | Region 7 |
| --- | --- | --- | --- | --- | --- | --- | --- |
| **ITEM 3** | 1. **A. Adjective; Categorially Ambiguous** | | | | | | |
|  | 동현이는 | 개가 | 무척 | **크다** | 여겨서 | 공원에 | 데려갔다. |
|  | Donghyeoni-neun | gae-ga | mucheok | **keu-da** | yeogy-eoseo | gongwon-e | deryeoga-ss-da. |
|  | Donghyun-Top | dog-NOM | very | big-PRS.IND | think-CAUSE | park-LOC | take-PST-DECL |
|  | “Donghyun thought the dog was very big, so he took it to the park.” | | | | | | |
|  | 1. **B. Verb; Categorially Ambiguous** | | | | | | |
|  | 동현이는 | 개가 | 무척 | **큰다** | 여겨서 | 공원에 | 데려갔다. |
|  | Donghyeoni-neun | gae-ga | mucheok | **keun-da** | yeogy-eoseo | gongwon-e | deryeoga-ss-da. |
|  | Donghyun-Top | dog-NOM | very | become.big- PRS.IND | think-CAUSE | park-LOC | take-PST-DECL |
|  | “Donghyun thought the dog was becoming very big, so he took it to the park.” | | | | | | |
|  | 1. **C. Adjective; Categorially Unambiguous** | | | | | | |
|  | 동현이는 | 개가 | 무척 | **많다** | 여겨서 | 공원에 | 데려갔다. |
|  | Donghyeoni-neun | gae-ga | mucheok | **mahn-ta** | yeogy-eoseo | gongwon-e | deryeoga-ss-da. |
|  | Donghyun-Top | dog-NOM | very | many- PRS.IND | think-CAUSE | park-LOC | take-PST-DECL |
|  | “Donghyun thought there were too many dogs, so he took them to the park.” | | | | | | |
|  | 1. **D. Verb; Categorially Unambiguous** | | | | | | |
|  | 동현이는 | 개가 | 무척 | **논다** | 여겨서 | 공원에 | 데려갔다. |
|  | Donghyeoni-neun | gae-ga | mucheok | **non-da** | yeogy-eoseo | gongwon-e | deryeoga-ss-da. |
|  | Donghyun-Top | dog-NOM | very | play- PRS.IND | think-CAUSE | park-LOC | take-PST-DECL |
|  | “Donghyun thought the dog was very playful, so he took it to the park.” | | | | | | |

|  | Region 1 | Region 2 | Region 3 | Region 4  (critical region) | Region 5  (spillover region) | Region 6  (spillover region 2) | Region 7 |
| --- | --- | --- | --- | --- | --- | --- | --- |
| **ITEM 4** | 1. **A. Adjective; Categorially Ambiguous** | | | | | | |
|  | 지훈이는 | 수염이 | 정말 | **길다** | 느껴서 | 거울을 | 보았다. |
|  | Jihuni-neun | suyeom-i | jeongmal | **gil-da** | neukk-yeoseo | geour-ul | bo-ass-da. |
|  | Jihun-Top | beard-NOM | really | long-PRS. IND | feel-CAUSE | mirror-ACC | look-PST-DECL |
|  | “Jihun felt that his beard was really long, so he looked in the mirror.” | | | | | | |
|  | 1. **B. Verb; Categorially Ambiguous** | | | | | | |
|  | 지훈이는 | 수염이 | 정말 | **긴다** | 느껴서 | 거울을 | 보았다. |
|  | Jihuni-neun | suyeom-i | jeongmal | **gin-da** | neukk-yeoseo | geour-ul | bo-ass-da. |
|  | Jihun-Top | beard-NOM | really | become.long-PRS. IND | feel-CAUSE | mirror-ACC | look-PST-DECL |
|  | “Jihun felt that his beard was becoming really long, so he looked in the mirror.” | | | | | | |
|  | 1. **C. Adjective; Categorially Unambiguous** | | | | | | |
|  | 지훈이는 | 수염이 | 정말 | **없다** | 느껴서 | 거울을 | 보았다. |
|  | Jihuni-neun | suyeom-i | jeongmal | **eobs-da** | neukk-yeoseo | geour-ul | bo-ass-da. |
|  | Jihun-Top | beard-NOM | really | not.be-PRS.IND | feel-CAUSE | mirror-ACC | look-PST-DECL |
|  | “Jihun felt that he really didn't have a beard, so he looked in the mirror.” | | | | | | |
|  | 1. **D. Verb; Categorially Unambiguous** | | | | | | |
|  | 지훈이는 | 수염이 | 정말 | **난다** | 느껴서 | 거울을 | 보았다. |
|  | Jihuni-neun | suyeom-i | jeongmal | **nan-da** | neukk-yeoseo | geour-ul | bo-ass-da. |
|  | Jihun-Top | beard-NOM | really | grow-PRS. IND | feel-CAUSE | mirror-ACC | look-PST-DECL |
|  | “Jihun felt that his beard was really growing (too many), so he looked in the mirror.” | | | | | | |

|  | Region 1 | Region 2 | Region 3 | Region 4  (critical region) | Region 5  (spillover region) | Region 6  (spillover region 2) | Region 7 |
| --- | --- | --- | --- | --- | --- | --- | --- |
| **ITEM 5** | 1. **A. Adjective; Categorially Ambiguous** | | | | | | |
|  | 동연이는 | 아침부터 | 항상 | **늦은** | 아내를 | 위해서 | 요리했다. |
|  | Dongyeoni-neun | achim-buteo | hangsang | **neuj-eun** | anae-reul | wihaeseo | yoriha-ess-da. |
|  | Dongyeon-Top | morning-FROM | always | late-PRS.ADN | wife-ACC | for | cook-PST-DECL |
|  | “Dongyeon cooked for his wife, who was always late in the morning.” | | | | | | |
|  | 1. **B. Verb; Categorially Ambiguous** | | | | | | |
|  | 동연이는 | 아침부터 | 항상 | **늦는** | 아내를 | 위해서 | 요리했다. |
|  | Dongyeoni-neun | achim-buteo | hangsang | **neun-neun** | anae-reul | wihaeseo | yoriha-ess-da. |
|  | Dongyeon-Top | morning-FROM | always | become.late-PRS.ADN | wife-ACC | for | cook-PST-DECL |
|  | “Dongyeon cooked for his wife, who was always running late in the morning.” | | | | | | |
|  | 1. **C. Adjective; Categorially Unambiguous** | | | | | | |
|  | 동연이는 | 아침부터 | 항상 | **예쁜** | 아내를 | 위해서 | 요리했다. |
|  | Dongyeoni-neun | achim-buteo | hangsang | **yepp-eun** | anae-reul | wihaeseo | yoriha-ess-da. |
|  | Dongyeon-Top | morning-FROM | always | pretty-PRS.ADN | wife-ACC | for | cook-PST-DECL |
|  | “Dongyeon cooked for his wife, who was always pretty in the morning.” | | | | | | |
|  | 1. **D. Verb; Categorially Unambiguous** | | | | | | |
|  | 동연이는 | 아침부터 | 항상 | **굶는** | 아내를 | 위해서 | 요리했다. |
|  | Dongyeoni-neun | achim-buteo | hangsang | **gum-neun** | anae-reul | wihaeseo | yoriha-ess-da. |
|  | Dongyeon-Top | morning-FROM | always | starve-PRS.ADN | wife-ACC | for | cook-PST-DECL |
|  | “Dongyeon cooked for his wife, who was always starving in the morning.” | | | | | | |

**4. Results of the follow-up self-paced reading experiment**

**Table 2. Statistical analyses of linear mixed model by the critical region (Region 4) and spillover region (Region 5) from the follow-up self-paced reading experiment.**

***Note*.** Asterisks (*) indicate significant differences across conditions (‘*’ indicates p<0.05, ‘**’ indicates p<0.01, ‘***’ indicates p<0.001).

|  | Estimate | SE | t-value | p-value |
| --- | --- | --- | --- | --- |
| Critical Region | | | | |
| (Intercept) | 6.12 | 0.05 | 131.90 |  |
| Category | 0.03 | 0.03 | 1.14 | 0.26 |
| Category Ambiguity | 0.01 | 0.02 | 0.45 | 0.65 |
| **Category × Category Ambiguity** | **0.08** | **0.04** | **2.18** | **0.03*** |
| Spillover Region 1 | | | | |
| (Intercept) | 6.16 | 0.05 | 131.82 |  |
| **Category** | **0.14** | **0.02** | **6.31** | **4.112e-10 ***** |
| Category Ambiguity | 0.03 | 0.02 | 1.25 | 0.21 |
| **Category × Category Ambiguity** | **0.10** | **0.04** | **2.28** | **0.02*** |

**Figure 2. Mean reading times at the critical region (Region 4) and spillover region (Region 5) for four conditions.**

**
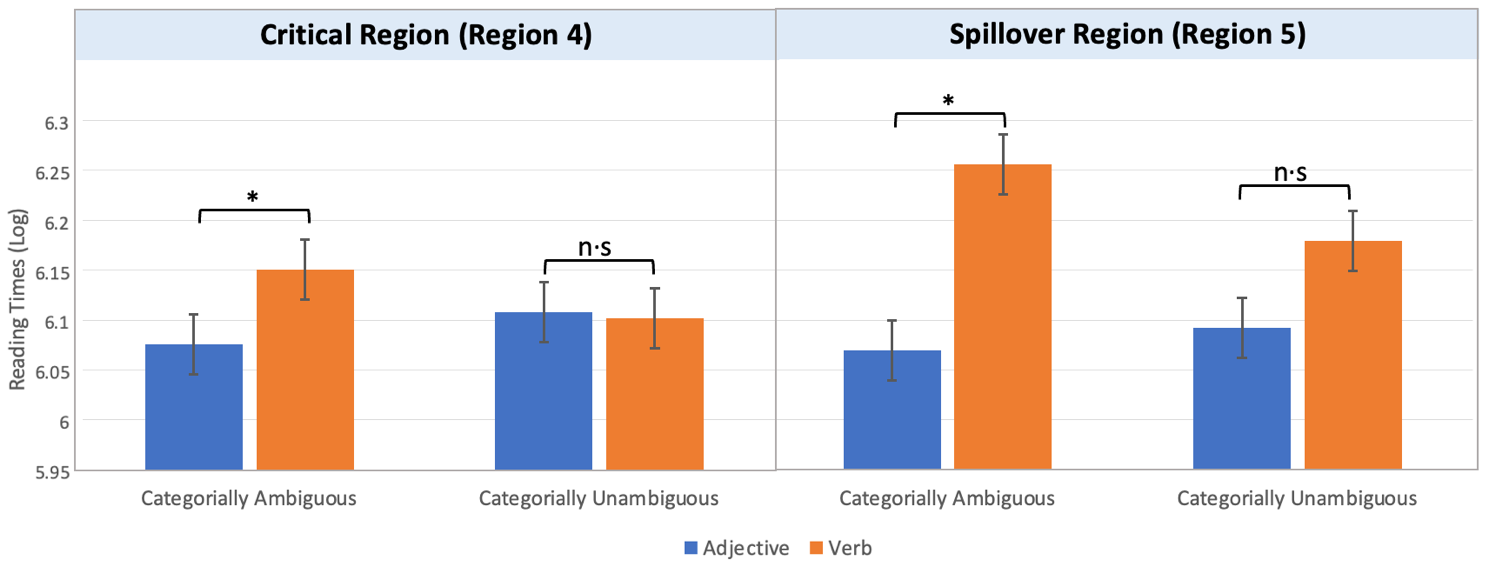
**

***Note*.** Asterisks (*) indicate significant differences across conditions (‘*’ indicates p<0.05 and ‘n·s’ indicates non-significant).
